# Preoperative Hypoalbuminemia as an Independent Predictor of Postoperative Acute Kidney Dysfunction After Cardiac Surgery

**DOI:** 10.1016/j.atssr.2026.01.005

**Published:** 2026-01-22

**Authors:** Joshua G. Crane, Aman Sinha, Toyokazu Endo, Michele Gallo, Siddharth Pahwa, Yash Vaidya, Mark S. Slaughter, Jaimin Trivedi

**Affiliations:** 1 Department of Cardiovascular and Thoracic Surgery, University of Louisville, Louisville, Kentucky; 2 School of Medicine, University of Louisville, Louisville, Kentucky

## Abstract

**Background:**

Postoperative acute renal failure associated with mortality after cardiac surgery. Whereas many variables result in postoperative renal failure, current literature lacks commentary on preoperative serum albumin optimization.

**Methods:**

We performed a retrospective review of a single adult cardiac surgery center database between 2021 and 2024. Patients were divided into 2 cohorts: preoperative hypoalbuminemia (<3.5 g/dL) and normal albuminemia (3.5-5.4 g/dL). Demographic and clinical characteristics were compared. The primary outcome was kidney injury or kidney failure, defined by the RIFLE (risk, injury, failure, loss of kidney function, end-stage kidney disease) criteria. Multivariable logistic regression evaluated the association of preoperative albumin level and postoperative kidney dysfunction.

**Results:**

There were 1673 patients who underwent cardiac surgery during 4 years, 444 (27%) with hypoalbuminemia and 1229 (63%) with normal albuminemia. The hypoalbuminemia group was younger (63 vs 65 years; *P* < .01) and less likely to be male (66% vs 71%; *P* = .04), with lower body mass index (28 kg/m^2^ vs 30 kg/m^2^; *P* < .01). Preoperative creatinine concentration (1.0 vs 1.0; *P* = .85) was comparable between groups. Postoperative kidney dysfunction was significantly greater in the hypoalbuminemia group (12% vs 5%; *P* < .01). Multivariable logistic regression found hypoalbuminemia to be an independent risk factor for acute kidney dysfunction after cardiac surgery (odds ratio, 1.8 [1.1-3.1]; *P* < .05).

**Conclusions:**

Hypoalbuminemia is significantly associated with postoperative acute kidney dysfunction in cardiac surgery patients. Preoperative optimization may decrease the risk of renal complications.

## Visual Abstract

**Figure:**
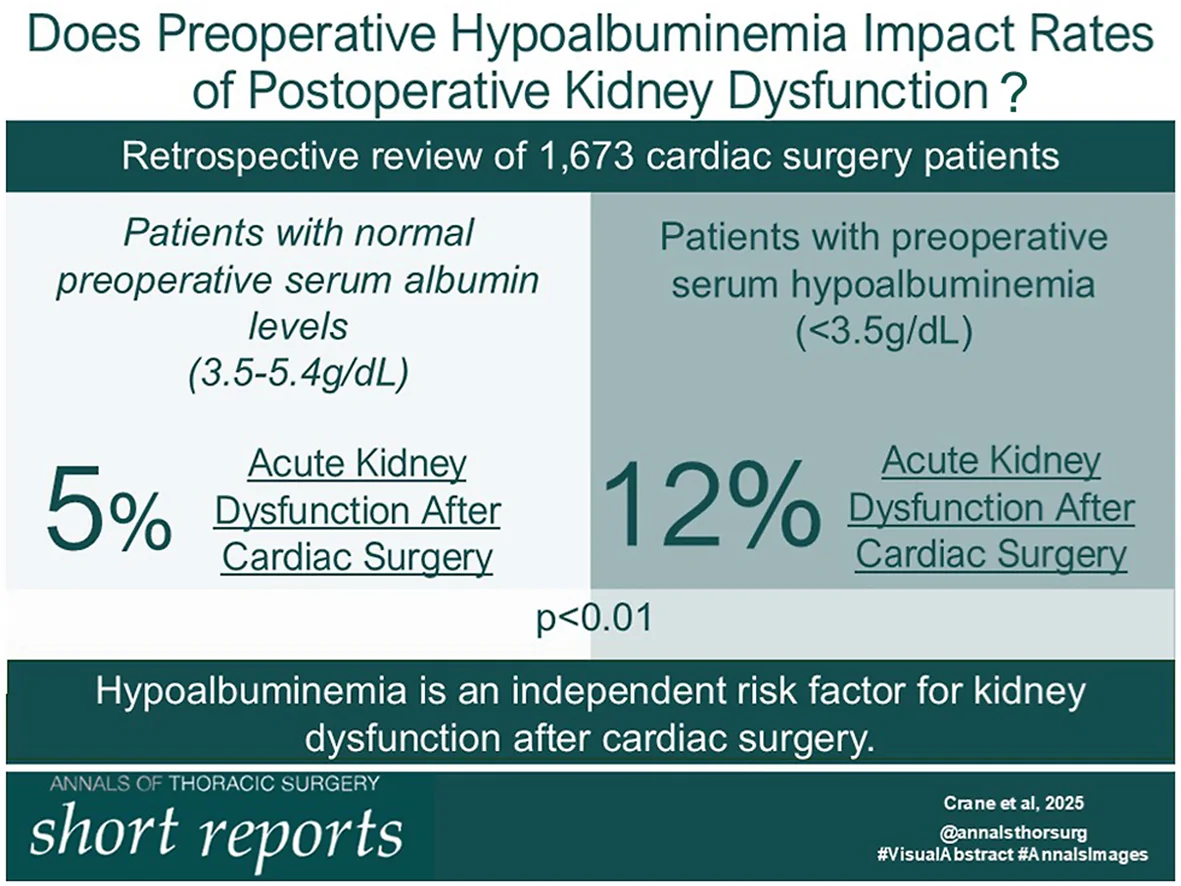

**In Short**

▪ Hypoalbuminemia is independently associated with the development of postoperative acute kidney dysfunction in cardiac surgery patients.
▪ Risk stratification for patients undergoing cardiac surgery should include an assessment of their preoperative serum albumin status.

The incidence of acute kidney injury after cardiac surgery ranges from 5% to 43%.^1^ Acute kidney injury after cardiac surgery is associated with increased risks of sepsis, atrial fibrillation, and surgical reexploration, often prolonging intensive care unit and hospital stays.^2^ Renal injury or failure, regardless of the need for renal replacement therapy, remains an independent risk factor for in-hospital mortality.^2^^,^^3^

Renal failure after cardiac surgery is multifactorial and influenced by various patient-specific and procedural risk factors. Newer studies have reported that perioperative fluid dynamics and resultant renal failure may be influenced by preoperative serum albumin levels.^4^ In this study, we aimed to evaluate the association between preoperative hypoalbuminemia and postoperative renal dysfunction, as defined by the RIFLE (risk, injury, failure, loss of kidney function, end-stage kidney disease) criteria, in patients undergoing open cardiac surgery.

## Patients and Methods

We conducted a retrospective analysis of a single-center adult cardiac surgery database from 2021 to 2024. All patients aged ≥18 years who underwent open cardiac surgery were included in the study. Patients undergoing cardiac transplantation or mechanical circulatory support implantation and with history of preoperative dialysis were excluded. Patients were then separated into 2 groups on the basis of preoperative serum albumin concentration: hypoalbuminemia (<3.5 g/dL) and normal albuminemia (3.5-5.4 g/dL). The baseline characteristics between the study groups were analyzed. Univariate nonparametric tests (Kruskal-Wallis test for continuous variables and *χ*^2^ test for categorical variables) were used to evaluate differences between the study groups.

The RIFLE criteria define kidney injury as an increase in creatinine concentration by 200% to 300% from baseline and kidney failure as an increase in creatinine concentration >300% from baseline or a new requirement of dialysis. For this analysis, kidney injury and kidney failure after cardiac surgery were combined to define acute kidney dysfunction. Multivariable logistic regression was performed to evaluate risk-adjusted association between albumin levels and postoperative acute kidney dysfunction. All analyses were performed with SAS 9.4 software (SAS Institute) at a 95% confidence level. The data were presented as median (interquartile range) and number (percentage).

## Results

There were 1673 adult cardiac surgery patients who met criteria for the study; 444 (27%) patients had hypoalbuminemia and 1229 (73%) had normal albuminemia (Table 1). Patients with normal albuminemia were older (65 vs 63 years; *P* = .01) and more likely to be male (71% vs 66%; *P* = .04), with higher body mass index (30 kg/m^2^ vs 28 kg/m^2^; *P* < .01). Patients with normal albuminemia had a lower preoperative white blood cell count (7.4 vs 8.3 × 10^3^/μL) and higher hemoglobin level (13.7 vs 11.8 g/dL; *P* < .01) and were less likely to have severe chronic lung disease (4% vs 12%; *P* < .01). Patients with hypoalbuminemia had higher myocardial infarction (53% vs 42%; *P* < .01), cardiogenic shock (14% vs 5%; *P* < .01), and endocarditis (14% vs 2%; *P* < .01). There was no difference in preoperative creatinine concentration (1.0 vs 1.0 mg/dL; *P* = .85).

**Table 1:** Demographics and Clinical Characteristics by Albumin Level

Variable	Normal Albumin Level (n = 1229)	Hypoalbuminemia (n = 444)	*P* Value
Age, y	65 (57-71)	63 (54-70)	.01
Sex, male	71 (876)	66 (293)	.04
Body mass index, kg/m^2^	30 (26-35)	28 (24-33)	<.01
Race			
White	87 (1069)	82 (363)	
Black	10 (120)	16 (70)	
Other	3 (40)	2 (11)	.002
Creatinine, mg/dL	1.0 (0.8-1.1)	1.0 (0.7-1.3)	.85
WBC count, × 10^3^/μL	7.4 (6.1-9.2)	8.3 (6.3-10.4)	<.01
Hemoglobin, g/dL	13.7 (12.6-14.9)	11.8 (10.2-13.3)	<.01
Platelet count, × 10^3^/μL	217 (181-260)	224 (177-288)	.02
Diabetes mellitus	42 (518)	44 (196)	.54
Hypertension	94 (1150)	88 (390)	.003
Chronic lung disease			
Moderate	13 (165)	14 (60)	
Severe	4 (52)	12 (50)	<.01
Cerebrovascular disease	25 (309)	36 (160)	<.01
Ejection fraction, %	57 (47-61)	55 (43-60)	<.01
Myocardial infarction	42 (516)	53 (236)	<.01
Cardiogenic shock	5 (57)	14 (58)	<.01
Endocarditis	2 (24)	14 (63)	<.01
Previous cardiac intervention	35 (434)	32 (138)	.11
ACE inhibitor or ARB	19 (229)	12 (53)	.001
Beta blocker	59 (730)	49 (217)	.001
Inotropic agent	3 (31)	8 (36)	<.01
Nonelective operation	43 (528)	77.25 (343)	<.01
Cardiopulmonary bypass time, min	97 (76-122)	97 (80-124)	.24
Intraoperative blood products	43 (524)	63 (281)	<.01
CABG	68 (832)	71 (317)	.28
Valve surgery	40 (492)	38 (170)	.54

Categorical variables are presented as percentage (number). Continuous variables are presented as median (interquartile range).

ACE, angiotensin-converting enzyme; ARB, angiotensin II receptor blocker; CABG, coronary artery bypass grafting; WBC, white blood cell.

Patients with hypoalbuminemia had a lower ejection fraction (55% vs 57%; *P* < .01), were more likely receiving inotropic agents (8% vs 3%; *P* < .01), and required intraoperative blood products (63% vs 43%; *P* < .01). There was no difference in cardiopulmonary bypass time (97 vs 97 minutes; *P* = .24), coronary artery bypass grafting procedures (71% vs 68%; *P* = .28), or valve surgery (38% vs 40%; *P* = .54).

The occurrence of postoperative acute kidney dysfunction was higher in the hypoalbuminemia group (12% vs 5%; *P* < .01). When stratified by severity, patients with hypoalbuminemia had greater rates of risk (14% vs 10%), injury (4% vs 2%), and failure (8% vs 3%; *P* < .01; Table 2). The multivariable logistic regression model found hypoalbuminemia to be an independent risk factor for postoperative acute kidney dysfunction (odds ratio, 1.8 [1.1-3.1]; *P* < .05). In addition, albumin level as a continuous variable showed that higher albumin level was protective of acute kidney dysfunction (odds ratio, 0.52 [0.32-0.85]; *P* < .01; Supplemental Figure). Other significant predictors included cardiogenic shock, endocarditis, and intraoperative blood product transfusion, whereas angiotensin-converting enzyme inhibitor use was associated with lower odds of acute kidney dysfunction (Figure).

**Table 2:** Postoperative Kidney Dysfunction by Albumin Level

Variable	Normal Albumin Level (n = 1229)	Hypoalbuminemia (n = 444)	*P* Value
Postoperative kidney dysfunction (RIFLE)	5 (61)	12 (52)	<.01
None	85 (1040)	74 (330)	<.01
Risk	10 (128)	14 (62)	
Injury	2 (26)	4 (18)	
Failure	3 (35)	8 (34)	

Values are reported as percentage (number).

RIFLE, risk, injury, failure, loss of kidney function, end-stage kidney disease.

**Figure.**
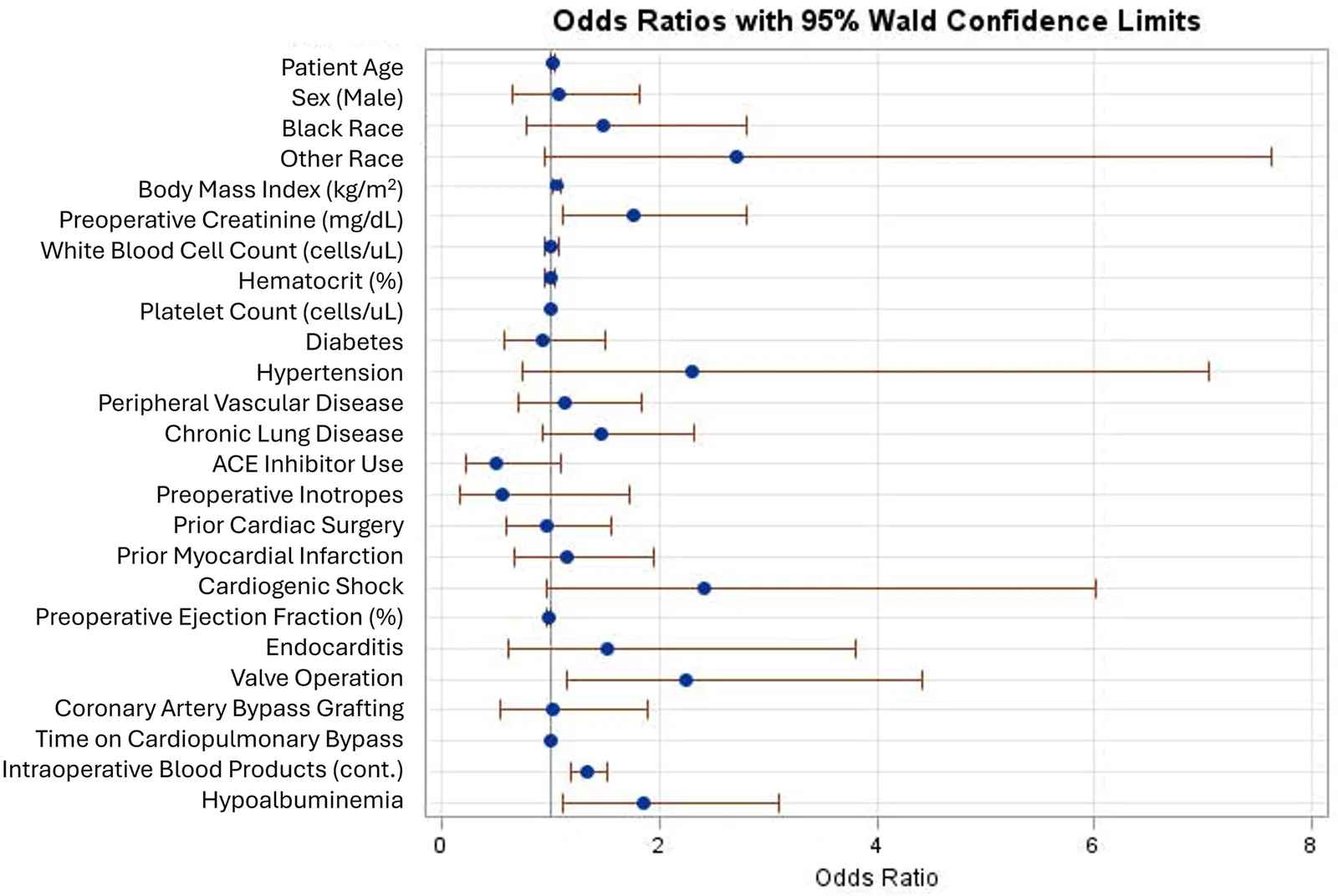
Multivariable logistic regression shows that hypoalbuminemia is an independent risk factor for postoperative acute kidney dysfunction (ACE, angiotensin-converting enzyme; cont., continuous).

## Comment

In patients with albumin levels <3.5 g/dL, risk of renal injury and failure after cardiac surgery is greater. Identifying modifiable preoperative factors, such as serum albumin levels, offers an avenue for risk stratification and intervention.

Albumin is a multifunctional serum protein that contributes to oncotic pressure, fluid homeostasis, and support of the endothelium of the renal capillaries. Hypoalbuminemia, chronically, is associated with decreasing kidney function and increasing rates of chronic kidney disease.^5^ Albumin’s function remains vital for capillary bed perfusion and endothelial integrity, underscoring the importance of albumin levels before cardiac surgery.

Low serum albumin levels are associated with other health conditions, such as frailty, sarcopenia, and overall malnutrition.^6^ These conditions may lead to a greater risk of postoperative renal failure in patients who are undergoing cardiac surgery.^7^ Albumin level is a critical identifier of patients who may be unfit for open surgery or who may need further risk stratification. ERAS (Enhanced Recovery After Surgery) protocols are often aimed at the preadmission testing of nutritional status because there is a known association between hypoalbuminemia and extended hospital stays, morbidity, and mortality.^8^ The impact of hypoalbuminemia on a patient’s renal status and sequelae make it an important factor to consider for risk stratification, and it should be considered a component of The Society of Thoracic Surgeons Adult Cardiac Surgery Database operative risk factor calculator.

In our cohort, patients with hypoalbuminemia differed substantially from those with normal albumin levels. They had lower body mass index, hemoglobin level, and ejection fraction as well as higher rates of cerebrovascular disease, endocarditis, and cardiogenic shock. These findings suggest that hypoalbuminemia often coexists with systemic illness, inflammation, and overall poor physiologic reserve. Importantly, some of these patients required more urgent or emergent cardiac operations, allowing limited preoperative correction of their albumin status. This study is limited by its retrospective nature.

In conclusion, preoperative hypoalbuminemia is an independent risk factor for the development of postoperative kidney dysfunction in patients who are undergoing cardiac surgery. Multifaceted optimization of a patient in the perioperative period of the cardiac procedure is required for the prevention of postoperative renal injury or failure. The significant relationship between renal failure and postoperative morbidity and mortality in cardiac surgery warrants investigation into causative factors. Preoperative albumin status should be considered a factor in The Society of Thoracic Surgeons Adult Cardiac Surgery Database operative risk factor calculator.
