# Supplementary material for: Preoperative Hypoalbuminemia as an Independent Predictor of Postoperative Acute Kidney Dysfunction After Cardiac Surgery
Source: Ann Thorac Surg Short Rep. 2026 Jan 22;4(3):1053–7. doi: 10.1016/j.atssr.2026.01.005 (PMC13555354; doi:10.1016/j.atssr.2026.01.005)
Supplement: Supplementary file 1 [file mmc1.docx]

Supplemental Figure 1
